# Perspectives of clinical pharmacists on the provision of pharmaceutical care through telepharmacy services during COVID-19 pandemic in Qatar: A focus group

**DOI:** 10.1371/journal.pone.0275627

**Published:** 2022-10-13

**Authors:** Eman Alhmoud, Dania Al Khiyami, Raja Barazi, Mohammed Saad, Ali Al-Omari, Ahmed Awaisu, Rasha El Enany, Moza Al Hail

**Affiliations:** 1 Pharmacy Department, Hamad Medical Corporation, Doha, Qatar; 2 Department of Clinical Pharmacy and Practice, College of Pharmacy, Qatar University, Doha, Qatar; Flinders University, AUSTRALIA

## Abstract

**Background:**

Coronavirus disease 2019 (COVID-19) pandemic created unprecedented pressures on healthcare systems and led to the widespread adoption of telepharmacy services, a practice that was not previously established in the state of Qatar.

**Objective:**

The -study aimed to explore clinical pharmacists’ (CPs) perspectives and experiences in utilizing telepharmacy for the provision of pharmaceutical care during the COVID-19 pandemic.

**Methods:**

A descriptive, qualitative approach using face-to-face focus group (FG) discussions was used. CPs across Hamad Medical Corporation (HMC) were purposively invited to participate in the study. FG discussions were audio-recorded, transcribed verbatim, and validated. Transcripts were analyzed using inductive thematic analysis. Recruitment continued until a saturation point was achieved.

**Results:**

We conducted five focus groups that included 23 CPs and led to seven themes. Overall, CPs reported inadequate preparedness for the practice of telepharmacy, which they perceived as challenging. The primary perceived benefits of telepharmacy were decreased infection exposure risk, improved quality of care, improved patients’ satisfaction, and enhanced workplace efficiency and productivity. The main highlighted risks of telepharmacy were related to threatened patient confidentiality, missed pharmaceutical care opportunities, and negatively impacted professional rapport with other healthcare providers; and the major perceived challenges were low digital health literacy, complex illnesses and medication regimens, lack of standardized protocols, and inadequacy of resources and cultural resistance for virtual care. Participants recommended standardization and training, resource allocation, and proper service promotion as potential facilitators of telepharmacy practice.

**Conclusion:**

The current study revealed that despite perceived barriers, pharmacists identified several benefits of telepharmacy and recommended potential facilitators that should be used to integrate and sustain the practice of telepharmacy in the future. Future studies should investigate the impact of telepharmacy on clinical pharmacy interventions and patient outcomes.

## Introduction

The pandemic of coronavirus disease 2019 (COVID-19) has resulted in unprecedented challenges to economics and healthcare systems [1]. The crisis demanded public health strategies to reduce the risk of COVID-19 transmission, preserve personal protective equipment, and accommodate patient surges at facilities while maintaining access to essential health services. Therefore, the practice of telemedicine was globally advocated [2,3].

Telemedicine is described as the utilization of information and communication technologies that include, audio or video equipment for the provision of healthcare services, by all healthcare professionals [4,5]. It allows distant two-way, interactive communication between a healthcare provider and a patient [5,6]. As defined by the National Association of Boards of Pharmacy (NABP) of the United States, telepharmacy is "the provision of pharmaceutical care through the use of telecommunications and information technologies to patients at a distance [7]. Previous studies have described the successful utilization of telepharmacy in rural, medically underserved communities for delivering various pharmaceutical services such as remote retailing and medication delivery, patient counseling, and clinical pharmacy reviews [7,8].

In Qatar, as the number of COVID-19 cases started to rise, the provision of virtual care through telemedicine and telepharmacy was widely adopted -to decrease infectious exposure risk and sustain the capabilities of the health system in the middle of COVID-19 peaks, and staff shortages. A practice that was rarely utilized before the pandemic. In-person (face-to-face) clinic visits were transformed into virtual visits, medication home delivery was launched, and clinical pharmacy services were provided remotely.

Thus, the aim of this study was to explore clinical pharmacists’ perceived benefits, risks, barriers, and facilitators related to the practice of telepharmacy during the COVID-19 pandemic.

## Methods

### Study design

A descriptive, qualitative approach using face-to-face focus group (FG) discussions was used in this study.

Focus groups take advantage of the interaction and in-depth discussions between participants to highlight their attitudes and understanding, enable the expression of a wider range of ideas and experiences and explore various perspectives arising from the debate within the group [9,10].

The study was conducted per the Consolidated Criteria for Reporting Qualitative Research (COREQ) checklist, a guide that promotes explicit and comprehensive reporting of qualitative studies [11].

### Participants

Clinical pharmacists across Hamad Medical Corporation (HMC), the principal public non-profit healthcare provider in the State of Qatar, were purposively selected to participate in the study.

Clinical pharmacy supervisors were excluded from this study to avoid the impact of power differentials, which could inhibit the participants from expressing their thoughts freely [12].

Recruitment continued until saturation was achieved and an agreement was made between the research team that no new concepts or ideas were emerging [13].

An email invite was sent to the candidates by the research team. The email included the research information sheet and a link to an electronic survey created via survey monkey to provide baseline demographics such as age, gender, and area of practice. The study was announced by emails and pharmacy leaders’ meetings across HMC.

### Focus group sessions

The face-to-face (60–90 minutes) focus groups took place within HMC premises. A researcher (DK or RB) moderated the discussion with the assistance of an observer (DK or RB), who helped in facilitation, when needed, and took field notes. Both researchers are registered clinical pharmacists (Doctor of Pharmacy (PharmD) holders) practicing in HMC with experience in moderating FG discussions Only the moderator, observer and participants were present.At the start of every FG, written informed consents were obtained from the participants.

To promote an organized flow of the discussion and in-depth exploration of opinions, a semi-structured facilitation guide developed from available literature in the field of telepharmacy, telemedicine and telehealth [14–17] was used.

The sessions were audio-recorded to ensure that all participants’ views were captured accurately. Participants were asked for approval of voice recording before each session. The recordings were stored on a password-protected computer within HMC. All audio recordings were transcribed verbatim by a researcher (AO). Subsequently, two authors independently (DA or RJ and EA) verified the transcripts for accuracy. Final revised transcripts were emailed back to the participants for comment and/or correction. Participants did not provide any feedback on the findings.

### Analysis

Data were analyzed by thematic content analysis. Two authors read each transcript, performed initial coding, clustered the codes, and defined emergent themes independently to increase the reliability of the data. Discrepancies between codes and themes generated by the researchers were subsequently discussed among the research team and resolved through consensus.

The study was approved by the Institutional Review Board (IRB) at HMC. (approval no: MRC-01-20-432).

## Results

Between November 2020 and February 2021, 25 clinical pharmacists (CPs) were invited to participate, of whom 23 attended five FG sessions (4–6 CPs per session). Two CPs refused to participate due to time constrains. On average, the focus groups lasted 64 minutes (range: 53 to 81 minutes). CPs had a median of 9 years of professional experience in HMC, and 56.5% were female. Most of the CPs (69.57%) had obtained board certifications. CPs practiced in different specialty areas that involved both in-patient and ambulatory settings, including COVID-19 facilities as illustrated in Table 1.

**Table 1:** Participants’ demographic and practice characteristics.

Characteristic	N = 24
Age (year)–median (IQR)	36.5 (26–48)
Number of years of experience (year)–median (IQR)	8 (2–15)
Number of years at HMC (year)–median (IQR)	9 (2–14)
Female gender–no. (%)	13 (54%)
Highest education—no. (%) Master’s degree Doctor of Pharmacy degree	10 (42%) 14 (58%)
Position—no. (%) Clinical pharmacist Clinical pharmacy specialist	17 (71%) 7 (29%)
Area of practice—no. (%) General medicine Critical care Emergency medicine Cardiology General pediatrics Infectious disease General surgery Oncology/hematology Others	9 (38%) 2 (8%) 2 (8%) 2 (8%) 2 (8%) 1 (4%) 1 (4%) 1 (4%) 4 (18%)

Seven themes emerged from the FG discussions, which were further subdivided into categories and subcategories. Table 2 summarizes information on the themes, categories, sub-categories, and selected quotes.

**Table 2:** Generated themes, sub-themes, and quotes.

Theme	Subtheme (category)	Quote
**Theme 1: Perceived meaning and scope of telepharmacy**	a. Interpretation of the meaning of telepharmacy	“Telepharmacy is caring for patients by pharmacists remotely through different methods of communication either phone or video conference or any other means of remote communication”- FG2, P4 “So, for me, telepharmacy is part of telemedicine”—FG4, P1 “I think telemedicine is more advanced than telepharmacy”—FG5, P5
	b. Technological platforms for providing care through telepharmacy	“The physicians in our setting were able to make video consultations, we didn’t do that in pharmacy, we just utilized the phone calls”—FG5, P5
	c. Spectrum of activities delivered via telepharmacy	“Reviewing the chart remotely, providing education to the patient and to the team and adjusting patient medications”- FG1, P5 “It’s the same idea, counseling, drug information inquiries, pharmacotherapy suggestions, medication availability, proper administration, the same interventions but through the phone” - FG4, P2
**Theme 2: Readiness and preparedness for practicing telepharmacy**	a. Awareness of existing guidelines and practices of telepharmacy	“There is an ACCP position paper or something like that”- FG1, P5 “I worked in a clinic where I did a lot of telepharmacy… I had a list of telepharmacy patients where I would call them, and I would actually make changes to their therapy. . .”—FG2, P4
	b. Receiving training for the implementation of telepharmacy	“Truly, we did not have any education about telepharmacy” -FG3, P2 We tried to take the model of telemedicine and apply it in telepharmacy but nothing systematic”- G5, P5
	c. Availability of operational support and resources for the provision of telepharmacy	“There was nothing in place to guide or to protocolize this process., No, not as per my information” -FG4, P2 “It wasn’t liked announced or standardized or planned”—FG5, P3 “I think, all in all, we did not have the preparedness to deal with the crisis across Qatar and the Middle East, we have no experience with such crisis. . .”—FG5, P4
**Theme 3: Experience with applying telepharmacy in practice**	a. Confidence in applying telepharmacy in practice	“You gain experience from different situations that you face with the patient or family members, so you get more confident by time”—FG5, P5 “I am confident, but I need resources” -FG2, P3
	b. Associated burdens of applying telepharmacy	“It was so difficult, so difficult, even sometimes physically and mentally exhausting, because you have many things to do, but you are limited” FG3, P2 “It was a huge, huge stress because there was no protocol in place to govern this process”—FG4, P2 “If in a normal situation, I don’t prefer remote coverage…no, face to face, you have to be there with patients…with the team”—FG1, P2 “For me, face-to-face coverage is much easier”-FG2, P
**Theme 4: Perceived benefits of providing care through telepharmacy**	**a. Patient-related telepharmacy benefits**	
	i. Reducing the risk of infectious exposure	“Patients were saved from coming to the hospital and being exposed during this time”—FG4, P5
	ii. Improving the quality of pharmaceutical care services (access to services, continuity of care, convenience, medication safety)	“Telepharmacy is very important especially in the era of COVID-19 because it was the only way to communicate with the patients”—FG3, P2 “We have seen patients who have lost follow-up for a year, but if you have the telepharmacy and someone to call and follow up with the patient, it would be better,” - FG2, P4 “Now the show is almost 100%, before COVID the show was around 70%”—FG2, P1 “Telepharmacy increases efficiency and improves patient care because the patient is just one phone call away” - FG2, P4 “During the pandemic, there was a lot of fake news, fake information. . .this was very important from the pharmacy perspective…to correct the information about the medications”- FG5, P1 “A lot of them are psychiatric and pediatric doctors, so you catch a lot of medication errors” - FG3, P5
	iii. Avoiding unnecessary costs and time	“SSome patients especially laborers are not able to come for the appointment just because of simple transportation fees… telepharmacy is free so there is a cost-benefit for the patients”—FG1, P4 “It’s more convenient, it saved the patients a lot of costs as well”—FG4, P5
	iv. Improving patients’ satisfaction	“I’ve seen a lot of patients who appreciate telepharmacy…they feel that even though I’m not going to the clinic, I’m not paying money, they still care and they’re still following me up”—FG2, P4 “Actually, too many patients have perceived the service as very efficient and effective and they like it better than coming to the clinic”- FG5, P4 “For patients, some of them were satisfied and some were not. It’s not because the quality of the service that was provided was different, that’s just because they don’t like the style of delivering the service” -FG2, P3
	**B. Health system-related telepharmacy benefits**	
	i. Decreasing risk of infectious exposure	“The first thing, it decreases the risk of infection transmission, so protect yourself and protect the patient and the team”—FG2, P2 “Of course, the safety as telepharmacy lessens the contact with the patient and other healthcare providers”—FG5, P2
	ii. Enhancing workplace efficiency and productivity	“You have to go to the floor, to your unit and then come back to be called again for one more education, so doing it through telepharmacy saved time”—FG1, P1 “I felt that it was a more effective round because, during the physical rounds, you got many interruptions…”—FG5, P2 “Two residents have done a research regarding the round and they found that there are at least two hours wasted… in nothing, only for going from one place to another…and then add to this the social issues”—FG3, P4 “Maybe I was better in documentation…. because there were less distractions”- FG4, P2 “We were able to maintain our capabilities for a longer period of time” -FG4, P1 “If we need to cover more than one area, for example, NICU, PICU, sometimes emergency at the same time, it saves time and can give a chance to focus on the more serious patients”—FG2, P2 “Maintain our service, this is the most important thing” -FG1, P3
	iii. Promoting effective communication between healthcare professionals	“I think in the outpatient, it improved the communication with physicians and nurses and even social workers…to get the best treatment plan and then to deliver it to the patient”- FG5, P5
**Theme 5: Perceived risks and drawbacks of providing care through telepharmacy**	a. Jeopardizing effective pharmaceutical care (lack of clinical assessment, acceptance, and timeliness of clinical pharmacists’ interventions,)	“I did an infectious disease telepharmacy, you would call the doctor and say, you know, we need to de-escalate to this antibiotic, and he says, oh my God, have you seen his leg? He has seen it and it’s horrible. I haven’t seen it. . . .”—FG2, P4 “If the team knows you, I think your intervention mostly will be accepted, if the team doesn’t know you, I think the acceptance rate could be lower”—FG2, P2
	b. Threatening s patient confidentiality	“I believe also the confidentiality of the patient was compromised, because sometimes you are in a shared office, and you have to talk with the patient over the phone like you do not have a private place to discuss issues with the patient ”—FG5, P2 “We don’t really have a method to identify patients, so you call and ask is this patient X? They will tell you yes. But how do you make sure that this is the patient, not the patient’s son, not the patient’s brother? So patient identification was really challenging”—FG2, P3
	c. Negatively impacting the working relationship between pharmacists and other healthcare professionals	“We worked hard to build the relationship with nursing…then we disappeared, so we lost that communication, and then we came back…to try to build it again, this was a disadvantage” - FG1, P4 “For me, it was a disadvantage, you totally lose the communication with health care professionals”—FG3, P2
	d. Demanding extra time and effort	“Sometimes it will take double the time that you used to take in the face-to-face educational session”—FG5, P2
	e. Decreasing patients’ satisfaction	“It was difficult especially if the patient is demanding that you come and see him or her like other professions, like nurse, like physician…they were around the patient, but you were not there, so they were questioning “why you are not coming?”—FG1, P4
**Theme 6: perceived barriers/challenges in implementing telepharmacy**	**a. Patient-related telepharmacy challenges**	
	i. Low digital health literacy	“You are utilizing technologies to communicate and also you are talking about health topics, so if the general population health literacy and technology literacy is low you will not be able to utilize the telepharmacy… Well even simple like messaging for informing some 60 years old, I will say someone with low health literacy and low technology literacy, simply telling him I will send you a picture of this through this platform, he will not be able even to download this platform”—FG1, P2
	ii. Complex patients and medication regimens	“If you have a complicated patient with multiple comorbidities and he has new medications started, you have to physically counsel, it will be very difficult as telepharmacy”–FG1, P1
	iii. Difficult communication	“When we see the patients physically sometimes, we can use the help of a translator colleague, a nurse, or some other colleagues, but over the phone, it’s almost impossible. So, the language barrier is a huge one” - FG4, P5. “You will lose all non-verbal communication, for example, clues for better understanding of the patient” - FG1, P4 “If you’re doing a chemotherapy education for the first time, you can express empathy in a face-to-face session, but it was very difficult over the phone”–FG5, P2 “You know, in the system… in Cerner, sometimes you can find six or seven numbers, contacting the first one, they transfer you to another number….and so on…of course, you will miss the patient”—FG2, P1 “Sometimes you don’t have the patient himself. One of the family will keep responding for his mobile. . . the mobile number that’s saved on the system. . .”—FG5, P1 “Some patients will be kind of not privileged by having high technology, their mobiles even are very very old without advanced technologies they will not be able to view videos or see PDFs or any kind of QR codes” - FG1, P4
	**b. Provider-related telepharmacy challenges**	
	i. Difficult communication	“WWhen you are trying to communicate to this specific patient you find yourself spending half an hour trying to reach the doctor trying to reach the nurse”–FG1, P5 “MMore than 60% of the communication will be lost if you don’t attend the round” -FG3, P2
	ii. Nonacceptance by other healthcare professionals	“You know, it’s a bad image…they were blaming you for not being there like them. They all have families, they all have risk factors, you know what I mean?” - FG4, P2 “The lack of communication between departments was a challenge. We had to explain to each one of the healthcare providers why we were not in the rounds”—FG1, P4 “For them to accept, we have to build a culture, this is something new to everybody, including the patients and the physicians… it is not accepted because it’s something new rather than it’s not efficient”—FG2, P3
	iii. Late or missing documentation	“I believe if you will get 100% full picture of the patient through face to face encounter with the team and the patient, actually through telepharmacy you will get two third or half that full picture because physicians will not have time to discuss over the phone as if they are face to face with you, so some of the data will be missing”—FG1, P2
	**c. System-related telepharmacy challenges**	
	i. LLack of standardization and training	“We thought about having video calls but again we were not familiar with that situation, we did not have training to do that…so it was difficult" - FG1, P4 “I think also one of the major barriers from my point of view is missing the standardization, every pharmacist is performing telepharmacy according to his way”—FG1, P3
	ii. Inadequacy of resources	“The challenges are huge…you need policies, you need hardware and software, you need running cost, you need to ensure the team itself is technologically empowered team”—FG4, P1 “We thought about having video calls but again we were not familiar with that situation, we did not have training to do that…so it was difficult"—FG1, P4 “There is a system, but the system is not open for other people to utilize it as it is needed. We don’t say there is no technology, no, there is a technology, but it is underutilized”—FG4, P4
**Theme 7: Suggested facilitators of successful implementing telepharmacy**	a. Standardization and training	“Have a pathway in place, this is the first thing. If you don’t have a pathway, you don’t have clear rights and the obligations for both the caller and receiver, nothing will work…things have to be standardized, it has to be very, very clear”—FG4, P2 “I think most of us should have telepharmacy training to be prepared more”—FG2, P2
	b. Collaboration and experience sharing	“I think collaboration is important to avoid this type of problems between different healthcare providers and to make a strategy about telemedicine, including telepharmacy, not to work in solos, working together is better I think”—FG5, P5 “First thing sharing experiences between others then we can have it standardized”—FG1, P5
	c. Resource allocation	“In my opinion, the first one is building a communication platform, you cannot just depend on a phone, we need something that’s secure and efficient in communication”—FG2, P4 "Educational material approved for the patients, platforms for communication”—FG1, P5 “Quiet place to make phone calls”—FG1, P4 “Can we also suggest a pharmacy website for HMC? So, patients can log in and put whatever they want. And then it is disseminated to the particular facility to provide their professional evidence-based answer” - FG5, P5
	d. Advertisement and promotion	“IInforming the patients on large scale, on Facebook, HMC website, Instagram that we are having this service. . . the community will have expectations or that they know that this service is there”—FG1, P2 “AAdvertisement should be there, not only for the public, it should be actually first for the physicians and nurses and then once you fix that part, you can go for the public”—FG4, P2

### Theme 1: Perceived meaning and scope of telepharmacy

The participants shared similar thoughts about the definition and scope of telepharmacy. They viewed telepharmacy as an alternative means of communication with patients and healthcare providers for the provision of pharmaceutical services when direct (face-to-face) contact is unachievable.

“*Telepharmacy is caring for patients by pharmacists remotely through different methods of communication either phone or video conference or any other means of remote communication*”- FG2, P4

One participant considered telepharmacy a part of telemedicine, whereas another believed that telemedicine is more advanced.

*“So*, *for me*, *telepharmacy is part of telemedicine”—*FG4, P1

*“I think telemedicine is more advanced than telepharmacy*”*—*FG5, P5

As they described the perceived meaning of telepharmacy, participants touched on available telepharmacy technologies, which included telephone calls, video calls (e.g., Zoom calls), text messages, and other communication platforms (e.g. Microsoft Teams). Of these, the telephone was the most commonly used modality as reported by some participants.

*“The physicians in our setting were able to make video consultations*, *we didn’t do that in pharmacy*, *we just utilized the phone calls*”*—*FG5, P5

Participants described telepharmacy activities provided during the pandemic as routine pharmaceutical services that are typically delivered through direct communications. These include remote medical chart review and subsequent communication of therapeutic recommendations to the healthcare team, medication reconciliation, patient education, and provision of drug information. One participant mentioned “*It’s the same idea*, *counseling*, *drug information inquiries*, *pharmacotherapy suggestions*, *medication availability*, *proper administration*, *the same interventions but through the phone*”

- FG4, P2

### Theme 2: Readiness and preparedness for practicing telepharmacy

The discussants were not aware of any existing practice guidelines for telepharmacy. Few participants knew about the American Society of Health-System Pharmacists (ASHP) s’ and the American College of Clinical Pharmacy (ACCP)’s position statements on telepharmacy.

Only one pharmacist had prior telepharmacy experience and commented that:

“*I worked in a clinic where I did a lot of telepharmacy… I had a list of telepharmacy patients where I would call them*, *and I would actually make changes to their therapy*…”—FG2, P4

Apart from personal efforts and some literature searches, none of the participants received formal telepharmacy education or training.

*“Truly*, *we did not have any education about telepharmacy”—*FG3, P2

Moreover, participants reported a lack of local supporting telepharmacy guidelines or policies. One participant commented: “*There was nothing in place to guide or to protocolize this process*. *No*, *not as per my information*”—FG4, P2

The participants also spoke about the inadequacy of telepharmacy resources, which is an identified challenge that will be further described under theme 6. One pharmacist related this to limited crisis preparedness in general: “*I think all in all*, *we did not have the preparedness to deal with the crisis across Qatar and the Middle East*, *we have no experience with such crisis*…”—FG5, P4

### Theme 3: Experience with applying telepharmacy in practice

Most participants reported being confident or very confident in applying telepharmacy. They expressed that their confidence increased as they acquired competence through clinical experience.

“*You gain experience from different situations that you face with the patient or family members*, *so you get more confident by time*”—FG5, P5

Many participants, however, described their experience with telepharmacy as being very difficult and challenging. They considered it an excessive burden that can lead to burnout. This was attributed to several challenges identified in theme 6. A participant remarked: “*It was a huge*, *huge stress because there was no protocol in place to govern this process*”—FG4, P2

Almost all participants favored in-person pharmaceutical services over telepharmacy.

*“If in a normal situation*, *I don’t prefer remote coverage…no*, *face to face*, *you have to be there with patients…with the team*”*—*FG1, P2

### Theme 4: Perceived benefits of providing care through telepharmacy

#### Patient-related telepharmacy benefits

Decreasing the spread of COVID-19 by limiting patients’ potential infectious exposures was perceived as the major benefit of telepharmacy as asserted by one participant *“Patients were saved from coming to the hospital and being exposed during this time”—*FG4, P5

Participants also highlighted how telepharmacy improved patients’ access to pharmaceutical care compared to in-person visits and maintained the continuity of care during the pandemic.

*“Telepharmacy is very important especially in the era of COVID-19 because it was the only way to communicate with the patients*”*—*FG3, P2

“*We have seen patients who have lost follow-up for a year*, *but if you have the telepharmacy and someone to call and follow up with the patient*, *it would be better*,”—FG2, P4

Improved access to care was evident by the improvement in the clinics’ show rate, according to one pharmacist “*Now the show is almost 100%*, *before COVID-19 it was around 70%*”—FG2, P1

Better access was attributed to convenience, cost, and time saving. A participant explained *“some patients especially laborers are not able to come for the appointment just because of simple transportation fees… telepharmacy is free so there is a cost-benefit for the patients*”*—*FG1, P4

Improved patients’ access to trusted health and drug information resources during the times of uncertainty and infodemic surrounding the pandemic was another benefit of telepharmacy as described by one participant: “During *the pandemic*, *there was a lot of fake news*, *fake information*…*this was very important from the pharmacy perspective*…*to correct the information about the medications*”- FG5, P1

Moreover, CPs emphasized the value of remote clinical pharmacy services in preventing several medication errors and enhancing patient safety during COVID-19 patients’ surge and deployment of doctors from different specialties to provide care.

“*A lot of them are psychiatric and pediatric doctors*, *so you catch a lot of medication errors*”

- FG3, P5

Many participants believed that telepharmacy improved patients’ satisfaction with pharmacy services.

“*I’ve seen a lot of patients who appreciate telepharmacy…they feel that even though I’m not going to the clinic*, *I’m not paying money*, *they still care and they’re still following me up*”

- FG2, P4

“*Actually*, *too many patients have perceived the service as very efficient and effective and they like it better than coming to the clinic*”- FG5, P4

However, there was a divergence of opinion on the level of patients’ satisfaction as some CPs mentioned patients’ dissatisfaction as well:

“*For patients*, *some of them were satisfied and some were not*. *It’s not because the quality of the service that was provided was different*, *that’s just because they don’t like the style of delivering the service*”—FG2, P3.

### Health system-related telepharmacy benefits

Limiting health care professionals’ infectious exposure risk was perceived as a major advantage of telepharmacy services.

“*The first thing*, *it decreases the risk of infection transmission*, *so protect yourself and protect the patient and the team*”—FG2, P2

Many participants noted that telepharmacy was more efficient than bedside rounds and led to enhanced work quality and productivity. They reported that telepharmacy provided an opportunity to stay focused, review a higher number of patients thoroughly and improve documentation as it avoided the travel time to patients’ wards and the round distractions.

“*You have to go to the floor*, *to your unit and then come back to be called again for one more education*, *so doing it through telepharmacy saved time*”—FG1, P1

“I felt that it was a more effective round because, during the physical rounds, you got many interruptions…”—FG5, P2

“Maybe I was better in documentation…. because there were less distractions”- FG4, P2

Participants also highlighted the role of telepharmacy in sustaining and expanding clinical pharmacy services to handle the COVID-19 patients’ surge during staff shortages. One pharmacist explained: *“We were able to maintain our capabilities for a longer period of time”—*FG4, P1

Another added: *“If we need to cover more than one area*, *for example*, *NICU*, *PICU*, *sometimes emergency at the same time*, *it saves time and can give a chance to focus on the more serious patients*”*—*FG2, P2

Some participants commented on improved communication among healthcare providers with telepharmacy, particularly in the ambulatory setting.

“*I think in the outpatient*, *it improved the communication with physicians and nurses and even social workers*…*to get the best treatment plan and then to deliver it to the patient*”- FG5, P5

However, most of the participants perceived telepharmacy as a threat to effective communication, particularly in acute care settings, which is addressed as a challenge in theme 6.

### Theme 5: Perceived risks and drawbacks of providing care through telepharmacy

Many participants were worried about the negative impact of telepharmacy on the quality of patient care as they highlighted the value of attending bedside multidisciplinary rounds on the development and execution of well-informed, effective, and timely clinical decisions. One participant remarked: *“I did an infectious disease telepharmacy*, *you would call the doctor and say*, *you know*, *we need to de-escalate to this antibiotic*, *and he says*, *oh my God*, *have you seen his leg*? *He has seen it and it’s horrible*. *I haven’t seen it*. . . .*”—*FG2, P4

Concerns about the loss of patient confidentiality were also raised. Potential confidentiality threats identified by participants included the inadequacy of private spaces to conduct virtual consults and the lack of robust patient identity verification systems.

*“I believe also the confidentiality of the patient was compromised because sometimes you are in a shared office*, *and you have to talk with the patient over the phone*, *like you do not have a private place to discuss issues with the patient*”*—*FG5, P2

*“We don’t really have a method to identify patients*, *so you call and ask is this patient X*? *They will tell you yes*. *But how do you make sure that this is the patient*, *not the patient’s son*, *not the patient’s brother*? *So patient identification was really challenging*”*—*FG2, P3

Participants also spoke about the negative impact imposed by telepharmacy on their professional rapport with other healthcare professionals who deprecated CPs’ absence from bedside patient care rounds. One participant explained “*We worked hard to build the relationship with nursing…then we disappeared*, *so we lost that communication*, *and then we came back*…*to try to build it again*, *this was a disadvantage*”

- FG1, P4

“*For me*, *it was a disadvantage*, *you totally lose the communication with health care professionals*”—FG3, P2

Many participants perceived a preestablished trusting relationship between CPs and physicians as an important determinant for the acceptance of their therapeutic recommendations. A participant commented: “*If the team knows you*, *I think your intervention mostly will be accepted*, *if the team doesn’t know you*, *I think the acceptance rate could be lower*”—FG2, P2

Some participants described telepharmacy as an extra burden, as they highlighted the considerable time and effort required to conduct a tele-consult.

“*Sometimes it will take double the time that you used to take in the face-to-face educational session*”—FG5, P2

Patients’ dissatisfaction with remote clinical pharmacy services was perceived as another telepharmacy drawback. Participants explained that some patients preferred direct over virtual consults, particularly in the inpatient setting. One pharmacist commented: “*It was difficult especially if the patient is demanding that you come and see him or her like other professions*, *like nurse*, *like physician…they were around the patient*, *but you were not there*, *so they were questioning* “*why you are not coming*?*”*—FG1, P4

### Theme 6: Barriers/Challenges in implementing telepharmacy

The sixth theme emerged from participants’ perceived challenges and barriers to the implementation of telepharmacy.

### Patient-related telepharmacy challenges

Participants identified patient’ characteristics that could impede the implementation of telepharmacy and compromise health outcomes.

Low digital health literacy was perceived as a major barrier to effective telepharmacy use. A participant commented: “*You are utilizing technologies to communicate and also you are talking about health topics*, *so if the general population health literacy and technology literacy is low you will not be able to utilize the telepharmacy*… *Well even simple like messaging for informing some 60 years old*, *I will say someone with low health literacy and low technology literacy*, *simply telling him I will send you a picture of this through this platform*, *he will not be able even to download this platform*”—FG1, P2

Participants also discussed the challenge of delivering virtual care to complex, multimorbid patients and patients receiving complex medications such as inhalers and injectables, where comprehensive in-person education was believed to be necessary to ensure safety.

*“If you have a complicated patient with multiple comorbidities and he has new medications started*, *you have to physically counsel*, *it will be very difficult as telepharmacy*” FG1, P1

*“If we have education for medications requiring explaining the technique…it is a major limitation to be honest*”—FG1, P5

Several patient-related communication challenges were highlighted. Of which, language barrier was the most emphasized. Many participants rely on interpreters (e.g., nurses) to communicate with non-Arabic or English proficient patients during in-person consultations, which was impractical virtually.

*“When we see the patients physically sometimes*, *we can use the help of a translator colleague*, *a nurse*, *or some other colleagues*, *but over the phone*, *it’s almost impossible*. *So*, *the language barrier is a huge one*”—FG4, P5

As telepharmacy relied vastly on telephone calls, the lack of nonverbal communication was identified as another barrier to effective communication.

*“You will lose all nonverbal communication*, *for example*, *clues for better understanding of the patient*”—FG1, P4

*“If you’re doing a chemotherapy education for the first time*, *you can express empathy in a face-to-face session*, *but it was very difficult over the phone*”–FG5, P2

Participants also described communication challenges related to the difficulty in reaching patients, such as outdated contact information in electronic health records (EHRs), patients not responding to the calls, and family interference.

“*You know*, *in the system… in Cerner*, *sometimes you can find six or seven numbers*, *contacting the first one*, *they transfer you to another number*….*and so on…of course*, *you will miss the patient*”—FG2, P1

Moreover, participants indicated that financial constraints could limit some patients’ accessibility to the internet or hi-tech devices required for tele consults.

“*Some patients will be kind of not privileged of having high technology*, *their mobiles even are very very old without advanced technologies*, *they will not be able to view videos or see PDFs or any kind of QR codes*”—FG1, P4

### Provider-related telepharmacy challenges

Participants identified a challenge in reaching some healthcare professionals to discuss patients’ updates and communicate therapeutic recommendations. This was further magnified when multiple specialties were involved in patient care.

“*When you are trying to communicate to this specific patient you find yourself spending half an hour trying to reach the doctor trying to reach the nurse*”–FG1, P5

Participants discussed at length the dissatisfaction and resistance expressed by other healthcare professionals toward transitioning to remote clinical pharmacy services, which negatively impacted pharmacists’ involvement in patient care. Many attributed this resistance to the lack of consistent communication about the service transition by the respective pharmacy leaders. Some participants even expressed feelings of embarrassment, stress, and self-defense as they frequently had to justify their absence from bedside rounds.

“*You know*, *it’s a bad image*…*they were blaming you for not being there like them*. *They all have families*, *they all have risk factors*, *you know what I mean*?”—FG4, P2

“*The lack of communication between departments was a challenge*. *We had to explain to each one of the healthcare providers why we were not in the rounds*”—FG1, P4

Participants also commented on the absence of telepharmacy culture, as another contributor to the encountered resistance.

“F*or them to accept*, *we have to build a culture*, *this is something new to everybody*, *including the patients and the physicians*… *it is not accepted because it’s something new rather than it’s not efficient*”—FG2, P3

Deficiencies in patients’ electronic records due to late or incomplete documentation was another perceived major challenge. As their care plan relied mainly on chart review, CPs were worried about missing vital patient information. One pharmacist explained:

“*I believe if you will get 100% full picture of the patient through face to face encounter with the team and the patient*, *actually through telepharmacy you will get two third or half that full picture because physicians will not have time to discuss over the phone as if they are face-to-face with you*, *so some of the data will be missing*”—FG1, P2

### System-related telepharmacy challenges

Most participants perceived insufficient operational and resource preparedness as a major challenge. Participants addressed the inadequacy of telepharmacy resources, and the lack of training, standardization, and consistent communication about the service transition as barriers toeffective telepharmacy implementation.

“*We thought about having video calls but again we were not familiar with that situation*, *we did not have training to do that*…*so it was difficult*"—FG1, P4

“*I think also one of the major barriers from my point of view is missing the standardization*, *every pharmacist is performing telepharmacy according to his way*”—FG1, P3

One participant commented that virtual care platforms were available, but CPs were not granted access to such platforms.

“*There is a system*, *but the system is not open for other people to utilize it as it is needed*. *We don’t say there is no technology*, *no*, *there is a technology*, *but it is underutilized*”—FG4, P4

### Theme 7: Suggested facilitators of successful implementation of telepharmacy

Most participants suggested standardizing the practice of telepharmacy through well-established protocols or pathways and training staff as major facilitators of successful telepharmacy implementation.

*“Have a pathway in place*, *this is the first thing*. *If you don’t have a pathway*, *you don’t have clear rights and the obligations for both the caller and receiver*, *nothing will work…things have to be standardized*, *it has to be very*, *very clear”—*FG4, P2

Few participants spoke about the value of collaboration and sharing telehealth experiences with other professions across the healthcare system.

“*I think collaboration is important to avoid this type of problems between different healthcare providers and to make a strategy about telemedicine*, *including telepharmacy*, *not to work in solos*, *working together is better I think*”—FG5, P5

*“First thing sharing experiences between others then we can have it standardized”—*FG1, P5

Participants also voiced the need for resources required to conduct teleconsultations effectively, such as dedicated spaces, information technology support, approved communication platforms, and educational materials.

*“In my opinion*, *the first one is building a communication platform*, *you cannot just depend on a phone*, *we need something that’s secure and efficient in communication”—*FG2, P4

"*Educational material approved for the patients*, *platforms for communication”—*FG1, P5

Some participants suggested the creation of a pharmacy portal, such as a hotline or a website to improve patients’ access to pharmaceutical services. One pharmacist commented

“*Can we also suggest a pharmacy website for HMC*? *So*, *patients can log in and put whatever they want*. *And then it is disseminated to the particular facility to provide their professional evidence-based answer*”—FG5, P5

Promotion of telepharmacy services through dedicated channels was also suggested to enhance awareness and cultural acceptance.

“*Informing the patients on large scale*, *on Facebook*, *HMC website*, *Instagram that we are having this service*… *the community will have expectations or that they know that this service is there*”—FG1, P2

“*Advertisement should be there*, *not only for the public*, *it should be actually first for the physicians and nurses and then once you fix that part*, *you can go for the public*”—FG4, P2

## Discussion

The current study explored clinical pharmacists’ perspectives on utilizing telepharmacy for the provision of pharmaceutical care during the COVID-19 pandemic in Qatar. We identified seven themes: perceived meaning and scope of telepharmacy, readiness and preparedness for practicing telepharmacy, experience with applying telepharmacy in practice, perceived benefits of providing care through telepharmacy, perceived risks and drawbacks of providing care through telepharmacy, barriers/challenges of implementing telepharmacy, and suggested facilitators of implementing telepharmacy.

Despite that most participants used telepharmacy for the first time during the COVID-19 pandemic, they reported using telepharmacy in a wide spectrum of activities across both inpatient and ambulatory settings. The diversity of the provided activities highlights the usefulness of telepharmacy in expanding pharmacy services during pandemics [18].

Participants perceived the practice of telepharmacy as challenging due to insufficient preparedness. Additionally, most participants were not aware of the available telepharmacy guidelines, a finding that is parallel to previous studies revealing limited knowledge about telemedicine among healthcare professionals [19,20]. Incorporating tele-education into the curricula of pharmacy schools and providing further telepharmacy exposure in the workplace were recently advocated by pharmacy professionals to ensure effective telepharmacy implementation [21,22].

The study participants used several platforms (e.g. Microsoft teams and zoom) to communicate with the healthcare teams and the patients. The phone was the most commonly used method, which is directly in line with the findings of a recent systematic review by Melton et al., where 76.3% of virtual clinical pharmacy services utilized phone-based interventions [23]. This finding was attributed to the lower equipment cost, greater availability, and ease of use.

Participants reported several advantages of telepharmacy, including reduced risk of patients’ and healthcare professionals’ exposure to infection as suggested by local and international health authorities [2]. Participants also appreciated that telepharmacy was a viable strategy to sustain pharmacy services at COVID-19 isolation facilities. A similar experience of providing pharmaceutical care to patients in COVID-19 cabin hospitals was reported in China during the early phase of the pandemic [24]. Additionally, better access to care, improved service efficiency, shorter waiting times, and better show rates at clinics were recognized as other telepharmacy advantages. This is consistent with previous literature, including pre-pandemic studies describing the benefits of telepharmacy [25–30].

While our study participants had mixed opinions on the impact of telepharmacy on patients’ satisfaction, previous literature indicated that telepharmacy enhances patient satisfaction [28,31,32]. A recent study from Iran revealed significant patients’ preference for telepharmacy services compared to in-person visits [33].

Participants reported several drawbacks of telepharmacy, including threatened patient confidentiality, which was previously reported with telemedicine [34]. The Concerns about loss of privacy and confidentiality were also highlighted in a recent review of telepharmacy practice during COVID-19, as many studies reported the inadequacy of teleconferencing platforms that are compliant with the Health Insurance Portability and Accountability Act (HIPAA) [35]. Moreover, some participants perceived the practice of telepharmacy as time-consuming. Similarly, devoting more time to conducting medication reviews has been suggested by Hanjani et al. [36]. Some study participants described the negative effect of remote services on timely patient assessment and execution of well-informed care plans in acute care settings (critical care units and emergency departments). Kosmisky et al., [37] described the use of technology in optimizing telepharmacy practice in the ICU. Interventions used included custom alerts that prompted patients review and electronic triage boards that flag pharmacists’ proposed interventions to the tele-ICU intensivists who review and respond to the interventions in real-time. In contrast, improvement in the quality of ambulatory pharmaceutical care was highlighted by some participants which is consistent with previous studies conducted in community health care settings [28,30]. Pharmacy leaders should consider this differential impact of telepharmacy in different health care settings when they utilize telepharmacy in practice.

Participants in our study discussed several challenges that hinder the effective implementation of telepharmacy. Low digital literacy and communication challenges were emphasized at patients’ level. Similarly, lack of digital access and low literacy were reported as major barriers to telepharmacy implementation in a recent review by Unni, et al. [35]. Killeen et al., proposed ways to overcome this challenge, which include opting for phone over video calls, at least for the first visit where proper patient education about the use of technology can be provided, and utilizing Wi-Fi video in place of cellular data to avoid unnecessary costs [38].

Another reported challenge was the lack of acceptance of telepharmacy by other health care providers and—patients. Similarly, Kosmisky et al. reported obtaining buy-in from bedside providers and adapting to intensivists’ preferences as barriers and challenges of telepharmacy implementation in an ICU setting in the US [37]. Moreover, Predmore et al. demonstrated that more than half of the US adults favored in-person visits over video visits if out-of-pocket costs were excluded. Those who preferred virtual visits were younger, with higher income and prior experience with video visits [39]. In our study, participants reported the lack of proper communication about telepharmacy as a contributor to telepharmacy resistance. Previous studies highlighted the importance of raising the community’s awareness of the availability of services [40,41]. Factors that may influence patients’ acceptance of virtual care need to be further explored and considered.

At the workplace level, the absence of standardized protocols and workflows for telepharmacy was perceived as a major challenge. Regionally, a recent study from the Kingdom of Saudi Arabia reported the lack of a national policy and well-standardized practices and processes at healthcare organizational levels as challenges for telepharmacy implementation [42].

Resource inadequacy was reported as another barrier, which was attributed to the emergent nature of the pandemic and the newness of such practice regionally. In a recent survey study by Muflih et al., only 26.1% of pharmacists in Jordan believed that their workplace is well equipped and prepared to launch telepharmacy [43].

Participants suggested several facilitators to overcome the aforementioned challenges, which mainly focused on adequate preparedness at various levels. These include the provision of practice governing protocols and pathways, staff training, and telepharmacy programs (hardware and software). Similarly, easier system implementation, better privacy and data protection, and simple to learn technology were reported as facilitators of the effective implementation of telepharmacy in a recent survey of Canadian pharmacists [22].

The current study has several strengths. It explored the perspectives of clinical pharmacists on the provision of pharmaceutical care through telepharmacy, a relatively new practice regionally with limited data about pharmacists’ experiences. We invited clinical pharmacists practicing through different specialties to obtain a representative sample and capture all possible experiences and perspectives. Additionally, we promoted participants’ ability to express their opinions freely and enrich the discussion by ensuring confidentiality and anonymity, excluding pharmacists in administrative positions and using a semi-structured discussion guide.

Our study, on the other hand, has some limitations. First, participants might have been conservative in expressing their opinions in the presence of coworkers. However, we minimized the risk of bias by allocating participants from different facilities to separate focus groups. Additionally, practicing during the COVID-19 pandemic might have affected participants’ perspectives toward telepharmacy. Nonetheless, the pandemic increased the need for telepharmacy and highlighted its role in improving patient outcomes.

## Conclusion

The current study revealed that despite perceived barriers, pharmacists identified several benefits of telepharmacy and recommended potential facilitators that should be utilized to integrate and sustain the practice of telepharmacy in the future. Future studies should investigate the impact of telepharmacy on clinical pharmacy interventions and patient outcomes.
